# Association Between Antihypertensive Treatment Discontinuation and the Development of Intracerebral Hemorrhage in Japanese Patients With Hypertension: The LIFE Study

**DOI:** 10.1161/JAHA.125.042523

**Published:** 2025-08-06

**Authors:** Kazuya Honda, Kenichi Goto, Megumi Maeda, Fumiko Murata, Haruhisa Fukuda

**Affiliations:** ^1^ Department of Health Sciences Kyushu University Graduate School of Medical Sciences Fukuoka Japan; ^2^ Department of Healthcare Administration and Management Kyushu University Graduate School of Medical Sciences Fukuoka Japan

**Keywords:** antihypertensive agents, hypertension, intracerebral hemorrhage, Japan, nested case–control study, risk factors, treatment discontinuation, Hypertension, Intracranial Hemorrhage

## Abstract

**Background:**

Hypertension is the principal risk factor for intracerebral hemorrhage (ICH), and blood pressure control is reliant on consistent adherence to antihypertensive treatment. This study examined the association between antihypertensive treatment discontinuation and ICH occurrence in Japanese patients with hypertension.

**Methods:**

This nested case–control study was conducted using claims data from the LIFE (Longevity Improvement & Fair Evidence) study from residents of 4 Japanese municipalities enrolled in public medical insurance or public assistance programs. The participants comprised people with hypertension between April and October 2017 and were followed up from November 2017 to March 2022 to identify ICH occurrence. Cases with ICH were matched with controls without ICH in a 1:10 ratio based on sex and age. Participants without any claims data for hypertension care for ≥4 months were regarded as discontinuing antihypertensive treatment. Conditional logistic regression was performed to examine the association between treatment discontinuation and ICH occurrence. Covariates included atrial fibrillation and flutter, diabetes, Charlson Comorbidity Index score, antithrombotic, and statin use.

**Results:**

Of 62 674 patients with hypertension, 5699 (9.1%) developed ICH during follow‐up. Participants who discontinued antihypertensive treatment had significantly higher odds for developing ICH (odds ratio, 3.77 [95% confidence interval, 3.43–4.14]; *P*<0.001) than treatment‐adherent participants. In addition, the male sex and younger age (<40 years) were significant risk factors for antihypertensive treatment discontinuation. Cases with ICH had significantly higher medical expenditure than controls without ICH.

**Conclusions:**

Antihypertensive treatment discontinuation significantly increased the risk of developing ICH in Japanese patients with hypertension. Regular medical visits and treatment adherence may help to prevent ICH development in these patients, thus reducing their clinical and economic burden.

**Nonstandard Abbreviations and Acronyms**
AFL: atrial flutter
CCI: Charlson Comorbidity Index
ICH: intracerebral hemorrhage
LIFE: Longevity Improvement & Fair Evidence
Statins: HMG‐CoA reductase inhibitors

**Clinical Perspective**

**What Is New?**

- In this nested case–control study using a large Japanese database of medical claims and other health‐related information, patients with hypertension who discontinued antihypertensive treatment for ≥4 months had a 3.8‐fold higher risk of intracerebral hemorrhage than those who remained adherent to treatment.
- Male sex and younger age (<40 years) were identified as significant risk factors for the discontinuation of antihypertensive treatment.

**What Are the Clinical Implications?**

- Discontinuation of antihypertensive treatment was significantly associated with an increased risk of intracerebral hemorrhage among Japanese patients with hypertension; regular medical follow‐up and adherence to antihypertensive treatment may help prevent intracerebral hemorrhage in this population, thereby mitigating the associated clinical and economic burden.

Intracerebral hemorrhage (ICH) is a serious condition with a high mortality rate and is often associated with long‐term disability in survivors.^1^, ^2^ Despite recent medical advances, the worldwide incidence of ICH has not decreased since the 1980s,^1^, ^2^ and outcomes of patients with ICH in Japan have not improved over the past 2 decades.^3^

Although older age, obesity, smoking, and alcohol consumption can increase the risk of ICH,^1^, ^2^, ^4^, ^5^ several epidemiological studies and meta‐analyses of randomized controlled trials have identified hypertension as the most important risk factor for ICH occurrence.^2^, ^5^, ^6^, ^7^ The risk of ICH is estimated to increase by at least 60% for every 10 mm Hg increase in systolic blood pressure,^8^ and a recent 10‐year study of 70 000 Japanese people determined that 57.1% of deaths from ICH were attributable to hypertension.^9^ Despite the high global prevalence of hypertension,^10^, ^11^ only 40% to 50% of hypertensive adults are diagnosed and treated, and less than half of these individuals have adequately controlled blood pressure levels.^11^, ^12^, ^13^ In addition, the incidence of ICH is generally higher in Asian populations than in people from other regions.^2^, ^6^ These studies emphasize the importance of adequate hypertension control in the prevention of ICH, especially in Japan and other Asian countries.

Lifestyle modification and consistent adherence to antihypertensive treatment are essential strategies for controlling blood pressure and preventing cardiovascular disease in hypertensive patients,^12^, ^14^ but these rely heavily on patient self‐management. Previous studies have shown that higher adherence to antihypertensive treatment is associated with a lower risk of hemorrhagic stroke.^15^ Similarly, a hospital‐based case–control study conducted in the 1990s found that the risk of ICH occurrence was significantly higher in individuals who had discontinued antihypertensive treatment than in treatment‐adherent patients.^16^ In the United States, the discontinuation rate of antihypertensive medication within 1 year of initiation was consistently found to be approximately 20% from 2007 to 2012.^17^ However, in the current medical landscape where numerous guidelines recommend strict hypertension management,^12^, ^14^, ^18^ few studies have examined the association between antihypertensive treatment discontinuation and ICH occurrence in real‐world settings.

This study aims to examine the association between antihypertensive treatment discontinuation and ICH occurrence in Japanese patients with hypertension, as well as to identify the potential factors contributing to antihypertensive treatment discontinuation. Due to concerns that health care costs and caregiver burden will continue to increase with the aging of Japan's population,^19^ we also examined the association between ICH occurrence and medical expenditure.

## METHODS

### Data Access and Availability

The data that support the findings of this study are not publicly available due to legal and administrative restrictions but may be provided upon reasonable request. For inquiries about the data sets used in this study, please contact the corresponding author.

### Study Design

This retrospective epidemiological study was conducted using a large database comprising medical claims and other health‐related data that are integrated at the individual level. In a typical prospective cohort study for a relatively rare target disease, it is necessary to follow a large cohort over a long period of time to ensure an adequate number of cases for analysis. To overcome this requirement, our study employed a nested case–control design, which enables highly accurate analyses while conserving resources by selecting a subset of a large cohort.^20^

This study was approved by the Kyushu University Institutional Review Board for Clinical Research (Approval number: 22114‐05). An opt‐out approach was used for participant consent, and the need for individual informed consent was waived due to the use of anonymized data.

### Data Source

Data were obtained from the LIFE (Longevity Improvement & Fair Evidence) Study, which is an ongoing database project established through agreements between Kyushu University (a Japanese national university) and various municipal governments.^21^ The LIFE Study was designed with the aim of facilitating cohort studies to provide evidence for increasing healthy life expectancy and reducing health disparities in Japan. At present, the LIFE Study collects various health‐related data (including health, medical, long‐term care, and administrative data) from over 30 municipalities. The data are anonymized before being collected and consolidated into data sets for research, and the different data types are linked at the individual level using research identification numbers. Due to differences in contractual agreements, there are variations in the data types and collection periods among the municipalities.

### Participants

For this study, we used data from 4 municipalities participating in the LIFE Study. The study data included records from people enrolled in National Health Insurance, the Latter‐Stage Older Persons Health Care System, and the Public Assistance System. National Health Insurance is a public medical insurance system covering the self‐employed, unemployed, irregularly employed, and primary sector workers aged ≤74 years. The Latter‐Stage Older Persons Health Care System is a public medical insurance system covering individuals aged ≥75 years. The Public Assistance System is a social welfare program that provides financial aid to low‐income households for living expenses, health care, housing, education, and other aspects of life. The study data set included 1 121 203 individual medical claims records.

All recorded diagnoses were identified using *International Classification of Diseases, Tenth Revision* (*ICD‐10*) codes in the claims data. Prescribed drugs were identified using the first 3 digits of the 12‐digit Japan Drug Classification codes. In addition, Anatomical Therapeutic Chemical classification codes were matched with the corresponding Japan Drug Classification codes to support identification and classification (Table S1).

The study was conducted using 5 years (60 months) of data from April 2017 to March 2022, which corresponded to the maximum overlap period in the data sets of the 4 target municipalities. The data were divided into 2 periods: Period I (April 2017 to October 2017; 7 months) and Period II (November 2017 to March 2022; 53 months).

The study participants comprised people with hypertension, who were identified as patients with recorded diagnoses of *ICD‐10* codes I10–I13 and I15 in Period I. Mortality cases from Period I and Period II were excluded from the analysis.

### Outcome of Interest

The study outcome was the occurrence of ICH (*ICD‐10* code: I61) during Period II. Recent validation studies in Denmark and Taiwan have reported high positive predictive values (83.7% and 84.4%, respectively) and sensitivities (98.6% and 97.5%, respectively) for using *ICD‐10* code I61 to identify ICH cases.^22^, ^23^ Their findings demonstrate the reliability of this code for ICH identification and add credibility to its use in our study. The first month in which patients had a recorded diagnosis of ICH in Period II was defined as their index date. Suspected diagnoses were not included when identifying cases with ICH.

Controls were selected from among the hypertensive patients without a recorded diagnosis of ICH in Period II. Cases were matched with controls in a 1:10 ratio based on sex and exact age in years using the index date of each case as the reference point. Matching was performed with replacement. Each control without ICH was assigned the same index date as their matched counterpart.

### Exposure

The study exposure was the discontinuation of antihypertensive treatment, which was defined as an absence of hypertension‐related diagnosis codes (ie, claims for antihypertension care) for ≥4 months during Periods I and II before the index date. For chronic diseases such as hypertension, it is common for outpatient and inpatient care to involve repeated prescriptions, often for extended periods of time. Under the Japanese health care system, patients typically have follow‐up visits and receive prescriptions at intervals of 1 to 3 months, with a legally mandated maximum prescription duration of 90 days. However, in real‐world clinical practice, patients who are prescribed a 90‐day supply of medications may occasionally return slightly later than scheduled due to forgetfulness or other nonemergency reasons. To reduce the risk of misclassifying such minor delays as treatment discontinuation, we used a threshold of ≥4 months (≥120 days) between prescription claims to identify “exposed” cases; these included patients whose treatment was discontinued by their own decision or the judgment of the health care provider. Conversely, patients with a recorded diagnosis of hypertension in their claims data at least once every 3 months during Periods I and II were classified as “unexposed.”

### Participant Characteristics

We analyzed the following participant characteristics: sex and age at the index date during Period II; and the total number of prescriptions, total number of outpatient visits, total number of hospitalizations, and total medical expenditure from the start of Period II to the index date. The number of prescriptions, number of outpatient visits, number of hospitalizations, and medical expenditure included those unrelated to antihypertensive treatment.

### Covariates

The study included the following covariates from Period I: atrial fibrillation and atrial flutter (AF/AFL), diabetes, total Charlson Comorbidity Index (CCI) score,^24^ prescriptions for antithrombotic agents, and prescriptions for the 3‐hydroxy‐3‐methylglutaryl coenzyme A reductase inhibitors (statins). AF/AFL was identified using *ICD‐10* code I48, and diabetes was identified using *ICD‐10* codes E10–E15. Suspected diagnoses of AF/AFL and diabetes were not included in the analysis. Antithrombotic prescriptions were identified using Japan Drug Classification codes 333 and 339, and statin prescriptions were identified using Japan Drug Classification code 218.

### Statistical Analysis

First, we descriptively analyzed the participant characteristics and covariates. The differences in these variables before the index date were compared between the cases with ICH and controls without ICH. Continuous variables were analyzed using Student's *t* test, and categorical variables were evaluated using the chi‐square test. When the expected frequency was <5, Fisher's exact test was used.

Next, we conducted conditional logistic regression analysis with ICH occurrence (outcome) as the dependent variable and antihypertensive treatment discontinuation (exposure) as the independent variable of interest. To adjust for potential confounders, the multivariable regression model incorporated AF/AFL, diabetes, CCI score, antithrombotic use, and statin use as covariates (Table S1). Using the regression analysis, we calculated the odds ratios (ORs) and 95% CIs of the independent variables. Additionally, we also conducted multivariable logistic regression analysis with antihypertensive treatment discontinuation (exposure) as the dependent variable. In this model, we examined the associations of various participant characteristics with antihypertensive treatment discontinuation. In the multivariable regression analyses, the Wald test was used to calculate the *P* values for the adjusted ORs.

All analyses were performed using RStudio ver. 2024.9.0.375 (Posit PBC, Boston, MA), with a 2‐sided significance level of *P*<0.05.

## RESULTS

### Participant Characteristics

**Figure 1:**
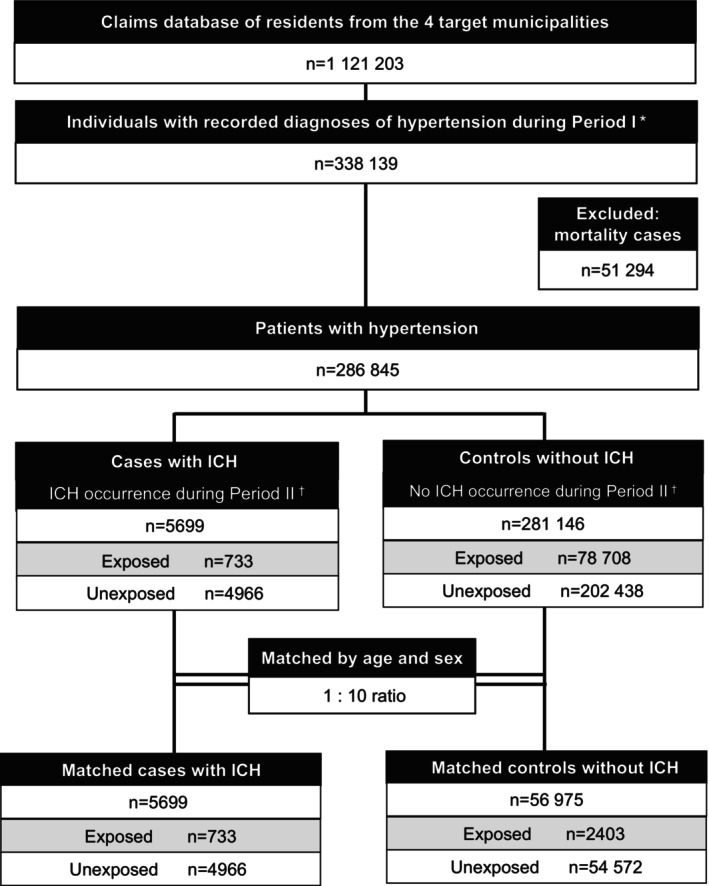
Flow chart of study cohort selection. Exposure refers to antihypertensive treatment discontinuation, which was defined as an absence of hypertension‐related care for ≥4 months. *Period I: April 2017 to October 2017 (7 months). ^†^Period II: November 2017 to March 2022 (53 months). ICH indicates intracerebral hemorrhage.

Figure 1 presents the study cohort selection process. The study was conducted using data from 62 674 patients with hypertension. The total number of participants who discontinued antihypertensive treatment (exposed group) during Periods I and II was 3136 (5.0%); among these, 733 were from the cases with ICH and 2403 were from the controls.

The participant characteristics are summarized in Table 1. Overall, the mean age of the participants was 73.7 years at the index date during Period II, and 50.3% were male. From the start of Period II to the index date, the mean number of hospitalizations was 1.23 (SD: ±3.97). The mean annual medical expenditure was 1 149 340 JPY (95 778 JPY/month), which was equivalent to approximately 10 448 USD (870 USD/month) at an exchange rate of 110 JPY per USD.

**Table 1:** Baseline Characteristics of the Cases With ICH and Controls Without ICH

Characteristics	Total	Cases with ICH	Controls without ICH	*P* value
	(n=62 674)	(n=5699)	(n=56 975)	
Demographics				
Age in y, mean±SD	73.7 (9.86)	73.6 (9.91)	73.7 (9.86)	0.902
Male sex	31 498 (50.3%)	2864 (49.7%)	28 634 (49.7%)	1.000
Age groups				
≥80 y	18 689 (29.8%)	1699 (29.8%)	16 990 (29.8%)	1.000
70–79 y	24 420 (39.0%)	2220 (39.0%)	22 200 (39.0%)	1.000
60–69 y	14 399 (23.0%)	1309 (23.0%)	13 090 (23.0%)	1.000
50–59 y	3685 (5.9%)	335 (5.9%)	3350 (5.9%)	1.000
40–49 y	1309 (2.1%)	119 (2.1%)	1190 (2.1%)	1.000
<40 y	172 (0.3%)	17 (0.3%)	155 (0.3%)	1.000
Comorbidities				
Atrial fibrillation/atrial flutter	4913 (7.8%)	620 (10.9%)	4293 (7.5%)	<0.001
Diabetes	25 638 (40.9%)	2175 (38.2%)	23 463 (41.2%)	<0.001
Charlson Comorbidity Index score, mean±SD	2.2 (1.73)	2.7 (1.78)	2.1 (1.71)	<0.001
Medications				
Antithrombotic use	23 751 (37.9%)	2589 (45.4%)	21 162 (37.3%)	<0.001
Statin use	28 009 (44.7%)	2247 (39.4%)	25 762 (45.2%)	<0.001
Health care use and expenditure				
Number of prescriptions, mean±SD	50.0 (88.97)	136.0 (150.64)	41.43 (74.99)	<0.001
Number of outpatient visits, mean±SD	30.4 (37.87)	78.5 (45.03)	25.53 (33.45)	<0.001
Number of hospitalizations, mean±SD	1.23 (3.97)	6.50 (9.05)	0.69 (2.44)	<0.001
Medical expenditure in yen, mean±SD	1 149 340 (3 057 854)	5 013 564 (6 487 698)	762 816 (2 105 534)	<0.001

All values are presented as n (%) unless indicated otherwise. ICH indicates intracerebral hemorrhage.

We identified 5699 participants (9.1%) who developed ICH during Period II. Cases with ICH had a significantly higher AF/AFL prevalence (*P*<0.001), higher CCI score (*P*<0.001), and a greater proportion of antithrombotic prescriptions (*P*<0.001) than the controls (Table 1). Additionally, cases with ICH exhibited significantly greater health care use, including more prescriptions (*P*<0.001), outpatient visits (*P*<0.001), hospitalizations (*P* < 0.001), and medical expenditure (*P*<0.001) than the controls.

### Association Between Antihypertensive Treatment Discontinuation and Intracerebral Hemorrhage

**Figure 2:**
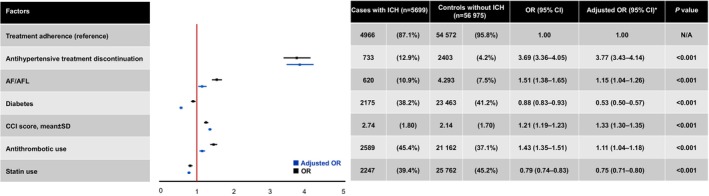
Conditional logistic regression analysis results of the factors associated with ICH development. All values are presented as n (%) unless indicated otherwise. *Adjusted for AF/AFL, diabetes, CCI score, antithrombotic use, and statin use. AF/AFL indicates atrial fibrillation and atrial flutter; CCI, Charlson Comorbidity Index; ICH, intracerebral hemorrhage; and OR, odds ratio.

The association between antihypertensive treatment discontinuation and ICH is shown in Figure 2. Antihypertensive treatment discontinuation was found to significantly increase the odds of developing ICH by approximately 3.8 times (OR, 3.77 [95% CI, 3.43–4.14]; *P*<0.001). Among the other independent variables, AF/AFL (OR, 1.15 [95% CI, 1.04–1.26]; *P*<0.001), CCI score (OR, 1.33 [95% CI, 1.30–1.35]; *P*<0.001), and antithrombotic use (OR, 1.11 [95% CI, 1.04–1.18]; *P*<0.001) were also significantly associated with increased odds of ICH. In contrast, diabetes (OR, 0.53 [95% CI, 0.50–0.57]; *P*<0.001) and statin use (OR, 0.75 [95% CI, 0.71–0.80]; *P*<0.001) were significantly associated with lower odds of ICH.

### Factors Associated With Antihypertensive Treatment Discontinuation

The factors associated with antihypertensive treatment discontinuation are shown in Table 2. Antihypertensive treatment discontinuation was positively associated with ICH occurrence (OR, 3.49 [95% CI, 3.20–3.82]; *P*<0.001), male sex (OR, 1.13 [95% CI, 1.04–1.20]; *P*<0.05), and younger age of <40 years (OR, 1.80 [95% CI, 1.04–2.90]; *P*<0.05). In contrast, antihypertensive treatment discontinuation was negatively associated with CCI score (OR, 0.92 [95% CI, 0.90–0.95]; *P*<0.001) and statin use (OR, 0.81 [95% CI, 0.75–0.88]; *P*<0.001).

**Table 2:** Factors Associated With Antihypertensive Treatment Discontinuation

Factors	Exposed*	Unexposed	OR (95% CI)	Adjusted OR†	*P* value
	(n=3136)	(n=59 538)		(95% CI)	
Outcome					
Controls without ICH (reference)	2403 (76.6%)	54 572 (91.7%)	1.00	1.00	
Cases with ICH	733 (23.4%)	4966 (8.3%)	3.35 (3.07–3.66)	3.49 (3.20–3.82)	<0.001
Demographics					
Age in y, mean±SD	73.1 (10.42)	73.7 (9.83)	1.00 (1.00–1.00)	0.99 (0.99–0.99)	0.330
Sex					
Female (reference)	1480 (47.2%)	29 696 (49.9%)	1.00	1.00	
Male	1656 (52.8%)	29 842 (50.1%)	1.00 (0.99–1.06)	1.13 (1.04–1.20)	<0.05
Age groups					
≥80 y (reference)	928 (29.6%)	17 761 (29.8%)	1.00	1.00	
70–79 y	1168 (37.3%)	23 252 (39.1%)	0.96 (0.88–1.05)	0.93 (0.85–1.02)	0.141
60–69 y	737 (23.5%)	13 662 (22.9%)	1.03 (0.93–1.14)	0.97 (0.88–1.08)	0.604
50–59 y	209 (6.7%)	3476 (5.8%)	1.15 (0.98–1.34)	1.06 (0.90–1.24)	0.469
40–49 y	77 (2.5%)	1232 (2.1%)	1.20 (0.93–1.51)	1.07 (0.83–1.35)	0.601
<40 y	17 (0.5%)	155 (0.3%)	2.10 (1.22–3.38)	1.80 (1.04–2.90)	<0.05
Comorbidities					
AF/AFL	264 (8.4%)	4649 (7.8%)	1.16 (1.01–1.32)	1.12 (0.98–1.29)	0.100
Diabetes	1206 (38.5%)	24 432 (41.0%)	0.99 (0.91–1.08)	1.09 (0.99–1.18)	0.080
CCI score, mean±SD	2.07 (1.67)	2.20 (1.73)	0.97 (0.94–1.00)	0.92 (0.90–0.95)	<0.001
Medications					
Antithrombotic use	1113 (35.5%)	22 638 (38.0%)	0.94 (0.86–1.02)	0.93 (0.85–1.01)	0.100
Statin use	1198 (38.2%)	26 811 (45.0%)	0.77 (0.72–0.83)	0.81 (0.75–0.88)	<0.001

All values are presented as n (%) unless indicated otherwise. AF/AFL indicates atrial fibrillation and atrial flutter; CCI, Charlson Comorbidity Index; ICH, intracerebral hemorrhage; and OR, odds ratio.

* Exposure refers to antihypertensive treatment discontinuation, which was defined as an absence of hypertension‐related care for ≥4 months.

^†^ Adjusted for AF/AFL, diabetes, CCI score, antithrombotic use, and statin use.

## DISCUSSION

This nested case–control study showed that the risk of ICH was 3.8 times higher in patients with hypertension who discontinued antihypertensive treatment for ≥4 months than treatment‐adherent patients. In addition, the male sex and younger age were significant risk factors for antihypertensive treatment discontinuation. Cases with ICH were associated with higher health care use and expenditure than controls. These findings may help to inform the development of targeted measures to prevent ICH in Japanese patients with hypertension, thus reducing their clinical and economic burden.

Asian populations, including Japan, generally have a higher incidence of ICH than other countries.^2^ Moreover, hypertension has been reported to be the principal risk factor for the development of ICH in Asians.^2^ Using data from the general Japanese population, Gotoh et al. showed that the incidence of ICH declined steeply from the 1960s to the 1970s but has since leveled off despite substantial improvements in hypertension control rates.^5^, ^12^ Similarly, a nationwide multicenter prospective cohort study found no clear improvements in outcomes for patients with hemorrhagic stroke in Japan between 2000 and 2019.^3^ These results indicate that the prevention and treatment of ICH remain important public health challenges.

The main finding of our study was the significant association between antihypertensive treatment discontinuation and ICH occurrence, with an adjusted OR of 3.8. As hypertension is the most important risk factor for ICH,^2^ it is reasonable to surmise that the increase in blood pressure following treatment discontinuation would elevate the risk of developing ICH. A previous Australian study similarly reported that antihypertensive treatment discontinuation increased the risk of developing ICH^16^; however, that study was conducted using a small, hospital‐based sample in the 1990s, when blood pressure targets for the prevention of cardiovascular disease were set higher than they are today. Our study using real‐world, large‐cohort data showed that antihypertensive treatment discontinuation significantly increased the odds of developing ICH in Japanese patients with hypertension.

In the present study, patients who discontinued their medications on their own and those whose medications were discontinued by their health care providers were collectively classified in the exposure category due to limitations in the claims data. This may have led to an underestimation of the association between antihypertensive treatment discontinuation and ICH occurrence, given that some previous studies have reported the achievement of sustained blood pressure control following withdrawal of antihypertensive medications, especially in older populations.^25^ In such instances—especially when discontinuation is carefully considered and managed by the treating physician—the associated risk of ICH may be relatively low. However, a meta‐analysis of randomized controlled trials that assessed the outcomes of antihypertensive medication discontinuation in older adults did not provide definitive evidence regarding the absence of increased cardiovascular risk.^26^ On the other hand, the same analysis reported a significant increase in blood pressure following treatment discontinuation,^26^ highlighting a potential consequence of withdrawing antihypertensive treatment.^27^ Indeed, a cohort study that followed older patients with hypertension over an average of 8.7 years reported a significantly higher risk of cardiovascular mortality among those who discontinued antihypertensive treatment, even when blood pressure remained below hypertensive thresholds.^28^ Together, these findings indicate that further research is needed to establish appropriate clinical criteria for the safe discontinuation of antihypertensive treatment; however, this issue lies beyond the scope of the present study.

Similar to previous studies,^29^, ^30^, ^31^ our study found that preexisting AF/AFL, CCI score, and concomitant antithrombotic use also significantly increased the odds of developing ICH. However, the adjusted ORs for these factors were much smaller than those for antihypertensive treatment discontinuation, highlighting the large adverse impact of treatment nonadherence on incident ICH. In contrast, preexisting diabetes significantly reduced the odds of developing ICH in our study, which was not consistent with the results of a previous meta‐analysis.^32^ Although we could not identify a definitive reason for this discrepancy, we note that the vast majority of our study participants would be Japanese, whereas 96% of participants in the meta‐analysis were from Europe, North America, and Australia. Therefore, ethnic and regional factors may have contributed to the difference in observations. In support of this hypothesis, 2 large long‐term observational studies in Japanese populations found no association between diabetes and ICH risk.^4^, ^33^

In our study cohort, concomitant statin use was associated with reduced odds of developing ICH. Although a post hoc analysis of the Stroke Prevention by Aggressive Reduction in Cholesterol Levels trial reported that statin treatment was associated with an increased risk of hemorrhagic stroke,^34^ a subsequent secondary analysis indicated that patients with hemorrhage were associated with inadequate blood pressure control.^35^ Because our study cohort was limited to patients with hypertension, we posit that most statin users would be on concomitant antihypertensive medication and therefore more likely to have adequate blood pressure control. Our findings are consistent with those of a recent analysis of Danish nationwide registries, which showed that concomitant statin and antihypertensive medication use was associated with a lower risk of ICH.^36^

Next, we found that antihypertensive treatment discontinuation was associated with the male sex and younger age. This was consistent with our previous study on medication adherence in Japanese patients with hypertension, which found poorer adherence to antihypertensive medication among young adults and men.^37^ Other studies have also reported that patients with discontinued or untreated hypertension are more likely to be male and younger (<50 years).^38^, ^39^ Notably, Ozawa et al. reported that the rate of untreated hypertension was as high as 79% among Japanese patients with hypertension aged <50 years who developed ICH.^40^ The observation that discontinued or untreated hypertension is more prevalent in younger patients has important implications because the impact of uncontrolled blood pressure on the risk of cardiovascular mortality is markedly amplified in younger people.^9^ Our findings may have clinical relevance not only to the Japanese population but also to other countries for 2 reasons: First, the incidence of ICH has been reported to be higher in men than in women in various regions, including the United States,^41^ Europe,^42^ and Japan.^4^ Second, higher rates of increase in ICH incidence have been reported in young (18–44 years) and middle‐aged (45–64 years) adults when compared with older adults (≥75 years) in the United States.^41^

According to data from the 2013 to 2014 US NHANES (National Health and Nutrition Examination Survey), only 33.7% of men aged 18 to 39 years with hypertension achieved adequate blood pressure control.^43^ A recent cross‐sectional study conducted in France further reported that approximately 30% of cases with hypertension in this age group were attributable to secondary hypertension.^44^ This finding may at least partly account for the suboptimal blood pressure control observed in young men with hypertension, as secondary hypertension is a well‐established contributor to resistant hypertension in this population.^45^ In such cases, discontinuation of antihypertensive treatment can result in a marked elevation in blood pressure, thereby increasing the risk of ICH. Therefore, effective strategies to encourage regular medical visits and consistent treatment adherence, especially in younger men with hypertension, are crucial to reducing the risk of ICH.

In our univariable analyses, cases with ICH showed higher CCI scores, health care use, and medical expenditure when compared with controls without ICH. These increases may have been affected by the higher prevalence of treatment discontinuation among the cases with ICH. Stroke and heart disease are known to be major contributors to increased medical expenditure.^46^ In particular, hospitalization for stroke results in higher medical expenditure, and in the case of residual disability, nonmedical costs (eg, caregiver costs and transportation costs) impose an additional financial burden.^46^, ^47^ Preventing antihypertensive treatment discontinuation is therefore expected not only to reduce ICH incidence but also contribute to a reduction in medical expenditure.

### Limitations

The limitations of this study are as follows. First, the study cohort was limited to residents of four municipalities, which may result in geographic, economic, and lifestyle biases. Second, when compared with patients who discontinued antihypertensive treatment, treatment‐adherent patients may also be more health conscious and possess better lifestyle habits that reduce their ICH risk. Accordingly, this could have introduced a healthy user bias into the study. Third, the study period spanned 5 years (2017–2022), which might not have been enough time to examine the incidence of ICH. This may have led to an underestimation of the impact of treatment discontinuation on ICH risk. Fourth, hypertension was identified based on diagnosis codes in claims data, which restricted the study cohort to patients who had already received medical intervention for hypertension. Fifth, the study data did not include indicators of disease severity, such as blood pressure levels, hypertension duration, types and quantities of prescribed antihypertensive medications, and whether the index ICH was a first or recurrent event. This limited the study's ability to fully assess the risk of developing ICH. Finally, we could not account for the effects of smoking, obesity, and alcohol consumption, which are known risk factors for ICH.^1^, ^2^, ^4^

## CONCLUSIONS

Our study showed that antihypertensive treatment discontinuation increased the odds of developing ICH by 3.8 times in Japanese patients with hypertension. This finding suggests that regular medical visits and treatment adherence are crucial to preventing ICH occurrence, which could also help to reduce health care use and expenditure. Because antihypertensive treatment discontinuation was more common in men and younger people, it may be prudent to design and implement effective strategies targeting these groups to encourage continued treatment adherence.

## Sources of Funding

This study was supported by a Grant‐in‐Aid for Scientific Research (KAKENHI) from the Japan Society for the Promotion of Science (Grant number: JP24K14194), a research grant from the Japan Agency for Medical Research and Development (Grant number: JP24ek0210174), and a research grant from the Japan Science and Technology (JST) Agency FOREST Program (Grant number: JPMJFR205J).

## Disclosures

None.

## Supporting information

Table S1
