# Supplementary material for: Association Between Antihypertensive Treatment Discontinuation and the Development of Intracerebral Hemorrhage in Japanese Patients With Hypertension: The LIFE Study
Source: J Am Heart Assoc. 2025 Aug 6;14(16):e042523. doi: 10.1161/JAHA.125.042523 (PMC12533608; doi:10.1161/JAHA.125.042523)
Supplement: Supplementary file 1 — Table S1 [file JAH3-14-e042523-s001.pdf]

SUPPLEMENTAL MATERIAL

Table S1. Definitions of Diseases and Treatments

	Definition		
	ICD-10 codes	JDC codes	ATC codes
**Target treatment and disease**			
Antihypertensive treatment	I10, I11, I12 I13, I15		
ICH	I61		
**Covariates**			
AF/AFL	I48		
DM	E10, E11, E12, E13, E14, E15		
Antithrombotics		333, 339	B01AA03, B01AB01, B01AB04, B01AB07, B01AB08, B01AB09, B01AE07, B01AF01, B01AF02, B01AF03, B01AX05, B01AX06, B01AX, B01AC04, B01AC05, B01AC06, B01AC19, B01AC22, B01AC23, B01AC24, B01AC30, B01AC56, B02BX07, B05BB01, B05CB02, B05CX10, C05BA01, L03AA, L03AX, L03AA02, L03AA10, L03AA13, L03AX16, V03AB37, V07AY
Statins		218	C10AB, C10AB01, C10AB02, C10AB05, C10AX, C10AD, C10AD01, C10AX02, C10AC01, C10AA03, C10AA01, C10AA04, C10AA05, C10AA08, C10AA07, C10AX09, C10AX06, C10AX12, C10BA06, C10AX13, C10AX14

AF/AFL indicates atrial fibrillation and atrial flutter; ATC, Anatomical Therapeutic Chemical; DM, diabetes mellitus; ICD-10, International Classification of Diseases, 10th Revision; ICH, intracerebral hemorrhage; and JDC, Japan Drug Classification.
